# CD20阴性/CD10阳性套细胞淋巴瘤（母细胞变异型）1例

**DOI:** 10.3760/cma.j.cn121090-20251223-00610

**Published:** 2026-06

**Authors:** 变变 乔, 雨欣 宁, 剑 郝, 春亮 史, 丹 刘, 楷 董, 耀方 张, 建梅 常, 方刚 任, 宏伟 王, 智芳 徐

**Affiliations:** 山西医科大学第二医院血液科实验室，血液病分子诊疗山西省重点实验室，太原 030001 Laboratory of Hematology, Second Hospital of Shanxi Medical University, The Key Laboratory of Molecular Diagnosis and Treatment of Hematological Diseases of Shanxi Province, Taiyuan 030001, China

患者，男性，68岁。因发现腹部肿物伴间断性腹痛于我院就诊。既往有高血压及脑梗死病史20年。查体：全身皮肤黏膜无黄染、出血点，左侧锁骨上窝可触及一约2.0 cm×1.0 cm肿大淋巴结。腹部未及明确包块。血常规：WBC 5.07×10^9^/L、淋巴细胞绝对计数0.12×10^9^/L、淋巴细胞百分比2.4％、HGB 97.0 g/L、PLT 149×10^9^/L、ANC 3.94×10^9^/L。生化：尿素15.80 mmol/L、肌酐108.00 µmol/L、LDH 593.00 U/L、β_2_微球蛋白3.61 mg/L。T细胞亚群：辅助性T细胞（Th）占19.3％，抑制性T细胞（Ts）占38.2％，Th/Ts比值为0.51，总T淋巴细胞占67.5％。自然杀伤细胞（NK细胞）占20.4％；B淋巴细胞占0.1％。胸部CT示左肺见长径0.5 cm微结节影，双侧胸腔少量积液，前纵隔见2.3 cm×1.3 cm实性结节，左侧内乳淋巴结肿大，短径约1.4 cm，其余未见明显异常。全腹盆腔CT提示左肾见长径3.4 cm圆形低密度影，腹盆腔及腹膜后多发肿大淋巴结，较大者约5.2 cm×9.6 cm，伴大量腹腔积液、肠系膜间隙模糊，膀胱壁增厚，其余实质脏器未见异常。床旁超声提示脾内见小结节，左肾见囊性无回声区，腹腔大量积液最深11.7 cm；颈部左侧、锁骨上窝多发异常肿大淋巴结，皮质增厚伴血流信号，其余浅表淋巴结未见明显异常。PET-CT示全身多部位多发肿大融合淋巴结伴^18^F-氟代脱氧葡萄糖高代谢，回盲部肠壁代谢增高提示受累，病灶肿瘤活性高，Deauville评分5分。

外周血细胞涂片可见中性中、晚幼粒细胞，分类100个白细胞可见幼红细胞7个；成熟红细胞大小不等；血小板成簇。骨髓细胞涂片显示骨髓增生极度活跃，粒系占11.5％，比例明显减低；红系占31.0％，以中晚幼红细胞为主，成熟红细胞大小不等；淋巴系占55.0％，幼稚样淋巴细胞占52.5％，细胞体积大，呈椭圆形或不规则形，胞质量极少，呈天蓝色，胞质内可见空泡，核多为圆形或椭圆形，染色质细腻，可见核仁（1～3个）。骨髓活检病理：送检骨髓穿刺组织，有效长度<1 cm，镜下：骨小梁间异型细胞局灶状增生浸润，细胞体积偏大，胞质量少，核略不规则、染色质较细腻；伴间质纤维化（MF-2）；其间少量三系造血细胞。颈部左侧淋巴结穿刺获取的淋巴组织切片苏木精-伊红（HE）染色结果显示正常结构消失，其间可见异型细胞弥漫浸润，细胞体积中等偏小，核圆形、不规则，部分可见小核仁。骨髓免疫组化：肿瘤细胞CD10、CD19、CD99、Cyclin D1、PAX-5阳性，CD5（局灶弱阳性），SOX-11（部分弱阳性），AE1/AE3、Bcl-2、Bcl-6、CD20、CD21、CD23、CD34、CD117、Mum-1、SOX-10、TDT阴性，Ki-67增殖指数为90％。造血组织CD3呈散在分布的T细胞阳性，CD15呈少量细胞阳性，CD42b每高倍镜视野下可见1至8个阳性细胞，CD68呈散在分布的细胞阳性，CD138呈少量散在分布的细胞阳性，E-cadherin呈簇状分布的细胞阳性，MPO呈少量细胞阳性。特殊染色：MASSON（+），网状纤维染色（MF-2），PAS（+），铁染色（+）。骨髓流式细胞术免疫分型：异常细胞占有核细胞61.64％，免疫表型为表达CD10、CD19、CD38、CD81、cyCD79a、CD123、cIgM,lambda；不表达cyCD3，CD5、CD7、CD13、CD20、cyCD22、CD23、CD33、CD34、CD66、CD117、CRLF2、FMC7、kappa、cyMPO、TDT。骨髓FISH检测结果显示CCND1/IGH双融合探针呈阳性，提示存在t（11;14）（q13;q32）易位。BCL2、BCL6及MYC断裂探针检测均为阴性，未见相关基因断裂或重排。最终诊断为套细胞淋巴瘤（母细胞变异型）ⅣA期，患者在明确诊断后未接受临床建议的治疗即离院，之后未能获得进一步随访信息。本例诊断关键依赖Cyclin D1强阳性、SOX11部分阳性及FISH检出CCND1/IGH重排。

